# Role of mustelids in the life-cycle of ixodid ticks and transmission cycles of four tick-borne pathogens

**DOI:** 10.1186/s13071-018-3126-8

**Published:** 2018-11-20

**Authors:** Tim R. Hofmeester, Aleksandra I. Krawczyk, Arieke Docters van Leeuwen, Manoj Fonville, Margriet G. E. Montizaan, Koen van den Berge, Jan Gouwy, Sanne C. Ruyts, Kris Verheyen, Hein Sprong

**Affiliations:** 1 Resource Ecology Group, Wageningen University, Wageningen, the Netherlands; 2 Present address: Department of Wildlife, Fish, and Environmental Studies, Swedish University of Agricultural Sciences, Skogsmarksgränd 17, 907 36 Umeå, Sweden; 3 Centre for Zoonoses and Environmental Microbiology, Centre for Infectious Disease Control, National Institute for Public Health and the Environment (RIVM), Bilthoven, the Netherlands; 4 Dutch Wildlife Health Centre (DWHC), Utrecht University, Utrecht, the Netherlands; 5 Research Institute for Nature and Forest (INBO), Geraardsbergen, Belgium; 6 Forest and Nature Lab, Department of Environment, Ghent University, Geraardsbergsesteenweg 267, 9090 Gontrode, Melle Belgium

**Keywords:** *Meles meles*, *Mustela putorius*, *Martes martes*, *Martes foina*, *Ixodes ricinus*, *Ixodes hexagonus*, *Anaplasma phagocytophilum*, *Borrelia burgdorferi* (*s.l.*), *Neoehrlichia mikurensis*, *Borrelia miyamotoi*

## Abstract

**Background:**

Elucidating which wildlife species significantly contribute to the maintenance of *Ixodes ricinus* populations and the enzootic cycles of the pathogens they transmit is imperative in understanding the driving forces behind the emergence of tick-borne diseases. Here, we aimed to quantify the relative contribution of four mustelid species in the life-cycles of *I. ricinus* and *Borrelia burgdorferi* (*sensu lato*) in forested areas and to investigate their role in the transmission of other tick-borne pathogens*.* Road-killed badgers, pine martens, stone martens and polecats were collected in Belgium and the Netherlands. Their organs and feeding ticks were tested for the presence of tick-borne pathogens.

**Results:**

*Ixodes hexagonus* and *I. ricinus* were found on half of the screened animals (*n* = 637). Pine martens had the highest *I. ricinus* burden, whereas polecats had the highest *I. hexagonus* burden. We detected DNA from *B. burgdorferi* (*s.l.*) and *Anaplasma phagocytophilum* in organs of all four mustelid species (*n* = 789), and *Neoehrlichia mikurensis* DNA was detected in all species, except badgers. DNA from *B. miyamotoi* was not detected in any of the investigated mustelids. From the 15 larvae of *I. ricinus* feeding on pine martens (*n* = 44), only one was positive for *B. miyamotoi* DNA, and all tested negative for *B. burgdorferi* (*s.l.*), *N. mikurensis* and *A. phagocytophilum*. The two feeding larvae from the investigated polecats (*n* = 364) and stone martens (*n* = 39) were negative for all four pathogens. The infection rate of *N. mikurensis* was higher in feeding nymphs collected from mustelids compared to questing nymphs, but not for *B. burgdorferi* (*s.l.*), *B. miyamotoi* or *A. phagocytophilum.*

**Conclusions:**

Although all stages of *I. ricinus* can be found on badgers, polecats, pine and stone martens, their relative contribution to the life-cycle of *I. ricinus* in forested areas is less than 1%. Consequently, the relative contribution of mustelids to the enzootic cycles of *I. ricinus*-borne pathogens is negligible, despite the presence of these pathogens in organs and feeding ticks. Interestingly, all four mustelid species carried all stages of *I. hexagonus*, potentially maintaining enzootic cycles of this tick species apart from the cycle involving hedgehogs as main host species.

**Electronic supplementary material:**

The online version of this article (10.1186/s13071-018-3126-8) contains supplementary material, which is available to authorized users.

## Background

Tick-borne diseases such as Lyme borreliosis (LB) and tick-borne encephalitis pose serious health concerns in Europe [1, 2]. Further geographical spread and long-lasting increases in the incidence of these two diseases have been observed in several European countries. Furthermore, diseases involving other tick-borne pathogens such as *Anaplasma phagocytophilum*, *Borrelia miyamotoi* and *Neoehrlichia mikurensis* are emerging or being (re)discovered [3]. Some of these pathogens are also of veterinary relevance, particularly *A. phagocytophilum*, which causes disease in dogs, horses and domesticated ruminants [4–8]. Understanding which factors drive population densities of ticks and the transmission cycles of these pathogens are important steps in assessing disease risk and formulating possible intervention strategies. One of these factors is the vertebrate community: wildlife and free ranging domestic animals act as feeding and propagation hosts of *I. ricinus* and as reservoir of tick-borne pathogens in nature areas [9, 10].

The causative agents of Lyme borreliosis are spirochetes belonging to the *Borrelia burgdorferi* (*sensu lato*) complex. At least five genospecies of *B. burgdorferi* (*s.l.*) have been shown to be pathogenic: *B. afzelii*, *B. garinii*, *B. burgdorferi* (*sensu stricto*), *B. bavariensis* and *B. spielmanii* [11, 12]*.* These genospecies are maintained through distinct enzootic cycles involving ticks and vertebrates acting as amplifying hosts [13]. For example, *B. afzelii* is mainly transmitted by small mammals, while *B. garinii* is mainly transmitted by birds [14, 15]. Even within genospecies, host species differ in their ability to transmit *B. burgdorferi* (*s.l.*) [16]. In Europe, these pathogens are predominantly transmitted by *Ixodes ricinus.* This generalist tick species has a three-host life-cycle; it depends on three vertebrate hosts for blood meals. Although *I. ricinus* utilizes a multitude of host species, these host species differ considerably in the number of ticks and the different life stages they feed [17]. Larvae mainly feed on small rodents and nymphs on multiple host species, whereas deer, most often roe deer (*Capreolus capreolus*), are the main hosts for adults [18, 19]. Finding out which host species contribute most in maintaining *I. ricinus* populations and transmitting *B. burgdorferi* (*s.l.*) is imperative in understanding the driving forces behind the enzootic cycles of this bacterial complex.

Both the number of ticks that a host successfully feeds and its ability to maintain and transmit a particular *B. burgdorferi* (*s.l.*) genospecies (i.e. reservoir competence) are important host characteristics [19–21]. The relative contribution to maintain *B. burgdorferi* (*s.l.*) in enzootic cycles, therefore, also depends on the density of the various vertebrate host species in the environment. Thus, the resulting distribution of *B. burgdorferi* (*s.l.*) in the questing ticks is a function of the densities of different host species, their capacity to feed ticks and to transmit the bacteria to those ticks. We previously performed an extensive systematic review to quantify the contribution of various vertebrate host species to feeding the different *I. ricinus* life stages, and transmitting *B. burgdorferi* (*s.l.*) genospecies to feeding larvae [18]. As the knowledge of the vertebrate assemblage in each environment is essential to perform these quantifications, we used data from the literature to approximate the vertebrate assemblage, including the most widespread vertebrate species occurring in most European forests. An important limitation of that review was that no, or only little, information was available for some widespread host species. In this study, we aimed to collect part of the missing data, namely that of several species of mustelids.

The family of mustelids (Mustelidae) consists of medium-sized to small carnivores, such as the Eurasian badger (*Meles meles*), the Eurasian otter (*Lutra lutra*), the European pine marten (*Martes martes*), the stone marten (*Martes foina*), the European polecat (*Mustela putorius*) and the common weasel (*Mustela nivalis*). Some mustelid species are closely bound to specific environments, such as the Eurasian otter to water, while others are habitat generalists occurring in most habitat types, such as the common weasel and the European polecat [23]. Of all European mustelid species, there are several that commonly occur in forested habitat and their ecotones and could therefore be important hosts for *I. ricinus*, namely the Eurasian badger, European pine marten, European polecat and stone marten [24]. These species are hereinafter referred to as badger, pine marten, polecat and stone marten.

Mustelids are often infested with *I. ricinus* [18, 25, 26], but can also carry *Ixodes hexagonus* [27] and other tick species [28]. *Ixodes hexagonus* is a nest-dwelling, three-host tick species. It parasitizes hedgehogs and carnivorous mammals from the families Canidae, Mustelidae and Felidae [29]. This tick species does not show any host preference between the adult and immature stages [27] and has been reported to bite humans as well [27, 30]. The vector capacity of *I. hexagonus* has been experimentally demonstrated for *B. burgdorferi* (*s.l.*) and tick-borne encephalitis virus [31–33], and has been suspected for other *I. ricinus-*borne pathogens as well, but without experimental proof [34–36].

In this study, we aimed to quantify the relative importance of four mustelid species hosting the different life stages of *I. ricinus.* We estimated the tick burden of these mustelids for other tick species and investigated their role in the transmission cycles of four tick-borne pathogens: *B. burgdorferi* (*s.l.*), *A. phagocytophilum*, *B. miyamotoi* and *N. mikurensis.* For *B. burgdorferi* (*s.l.*), we aimed to quantify the realized reservoir competence of these mustelid species, which is defined as the proportion of blood-fed larvae that become infected with a pathogen [37].

## Methods

### Sample collection in the Netherlands

**Fig. 1:**
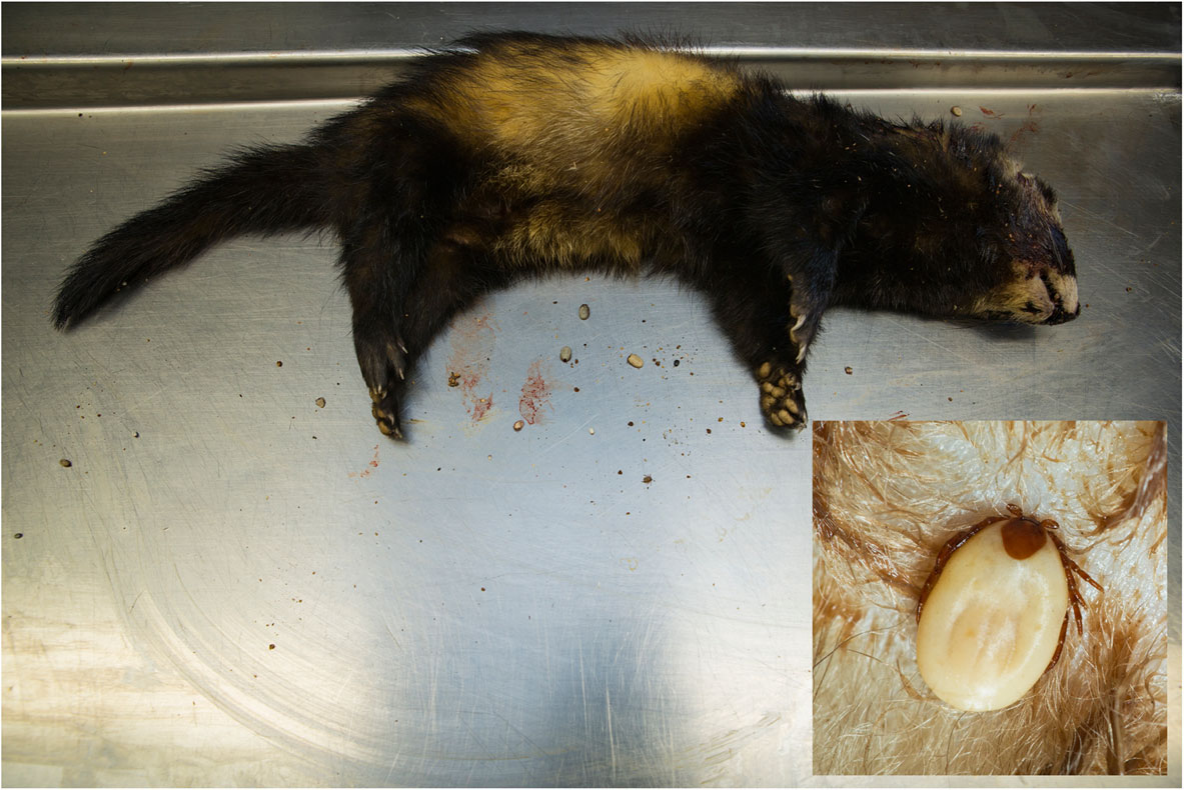
Road-killed polecat with fed ticks from collection bag. Inset: Feeding *I. hexagonus* female on pine marten

Mustelids killed by traffic or otherwise found dead were collected by volunteers throughout the Netherlands between 1995 and 2015. Animals were collected throughout the year in a range of habitats, from forest to agricultural fields and urbanized areas. Only animals that were perceived as recently killed and intact were taken into account. These animals were transported in a sealed plastic bag, ensuring that the ectoparasites that were still present on the animals were preserved (Figure 1). The animals were transferred to the Dutch Wildlife Health Centre (DWHC; 6 badgers), the Dutch Mammal Society or Wageningen Environmental Research (formerly Alterra). The animals sent to the DWHC were stored at 4 °C overnight and checked for ticks and dissected the following morning. All other animals were stored at -20 °C until further analysis. The animals were screened for ticks by thoroughly inspecting the skin and hairs of the head, neck, axillae, inguinal region and legs. The rest of the body was roughly inspected for ectoparasites, as was the contents of the transportation plastic bags. Ectoparasites were collected in a single tube with 70% ethanol per individual. These tubes were stored at -20 °C until further analysis. After screening for ectoparasites, tissue samples were taken from a subset of animals. Small parts of the liver and spleen were collected in 2 ml tubes when these organs were still intact after the impact of the vehicle. Ear biopsies were collected only from some of the animals. The dissection material and table were cleaned thoroughly between animals.

### Sample collection in Belgium

Analogous to the Netherlands, road-killed mustelids were collected since 1995 by a voluntary network (Marternetwerk; https://www.inbo.be/nl/marternetwerk) throughout Flanders, i.e. the northern part of Belgium, hereinafter referred to as Belgium. Systematic attention for ticks was given in the period until 2005. As pine martens were rare in Belgium until recent years [38], no specimens of this species were available for this study. Animals were collected throughout the year in a similar range of habitats as in the Netherlands. Dead animals were transferred in a sealed plastic bag to a local network freezer at -20 °C and later autopsied at the Research Institute for Nature and Forest (INBO). The screening of dead animals for ticks was performed as described above. The ectoparasites were collected in a small sealed plastic bag and stored at -20 °C. During the autopsy, liver samples were taken if possible and stored at -20 °C (no spleen samples or ear biopsies were taken). Not all animals from which a liver sample was taken were also screened for ticks, resulting in different sample sizes for tick burden and infection rate in organs. The dissection material and table were cleaned thoroughly between animals.

### DNA extraction, qPCR assays and sequencing procedures

DNA from ticks and tissues were extracted using the Qiagen DNeasy Blood and Tissue Kit (Qiagen, Venlo, the Netherlands) according to the manufacturer’s protocol [39]. For the detection of *B. burgdorferi* (*s.l.*) DNA, a duplex qPCR was used, based on the detection of fragments of the outer surface protein A (*ospA*) and flagellin genes [40]. A conventional PCR assay, targeting the 5S-23S intergenic spacer region (IGS), was performed for *B. burgdorferi* (*s.l.*) genospecies identification [41]. Conventional PCR assays were carried out in a Px2 thermal cycler (Thermo Electron Corporation, Breda, the Netherlands) and visualized on a 2% agarose gel. Both strands of PCR products were sequenced by BaseClear (Leiden, the Netherlands), according to the company’s protocol and using the same forward and reverse primers as in the conventional PCR [42]. BLAST analyses and in-house molecular epidemiological databases (Bionumerics 7.1 Applied Math, Sint-Martens-Latem, Belgium) were used to identify *B. burgdorferi* (*s.l.*) genospecies. These databases contain all our DNA sequences from (field) isolates, together with (reference) sequences from GenBank [11, 41]. For detection of *B. miyamotoi*, a qPCR assay was used that targets a region of the flagellin gene, specific for *B. miyamotoi* [43]. For detection of *A. phagocytophilum* and *N. mikurensis* DNA, a duplex qPCR assay was used, as described by Jahfari et al. [44, 45]. This qPCR assay targets specific regions of the major surface protein 2 gene (*msp2*) for *A. phagocytophilum* and a heat-shock protein gene (*groEL*) for *N. mikurensis*. All qPCR runs were carried out in a final volume of 20 μl, containing IQ Multiplex Powermix (Bio-Rad Laboratories, Hercules, CA, USA), 400 nM of primers and hydrolysis probes and 3 μl of DNA template. Conditions for PCR amplification were as follows: 95 °C for 5 min, 60 cycles at 95 °C for 5 s and 60 °C for 35 s, followed by a final incubation step at 37 °C for 20 s. We carried out qPCR assays on a LightCycler 480 instrument (Roche Diagnostics Nederland B.V, Almere, the Netherlands) and analysis was performed by the instrument’s software (release 1.5.1.62). Quantification cycle (C_q_) values were calculated using the second derivative method.

### Quantifying the relative importance of mustelids as hosts for *I. ricinus* and *B. burgdorferi* (*s.l.*)

We quantified the relative importance of the four mustelid species using the method described by Hofmeester et al. [18]. Briefly, we modeled a simplified host community based on species presence and densities in European forests, using the same species composition as [18] with the addition of the four mustelid species described in this study. For each of these species, we calculated the relative importance of the species in feeding *I. ricinus* ticks of the different life stages (${RI}_{l_i}$, ${RI}_{n_i}$, and ${RI}_{a_i}$ for the larval, nymphal and adult stage, respectively) as,

1 $$ {RI}_{l_i}=\frac{B_{l_i}{D}_i}{\sum_{j=1}^n{B}_{l_j}{D}_j} $$

where ${B}_{l_i}$ is the mean larval burden (no. of larvae per host individual) of species *i*, and *D*_*i*_ is the average density (km^-2^) in which species *i* occurs. To get a relative proportion, the total number of larvae fed by all species in the community (${\sum}_{j=1}^n{B}_{l_j}{D}_j$) is used. ${B}_{l_i}$ can be substituted by ${B}_{n_i}$ (the mean nymphal burden) or ${B}_{a_i}$ (the mean adult burden) to calculate ${RI}_{n_i}$ and ${RI}_{a_i}$, respectively.

Following Hofmeester et al. [18], we added the realized reservoir competence for *B. burgdorferi* (*s.l.*) (${RC}_{bb_i}$) to Equation 1 to estimate the relative importance of each species in infecting *I. ricinus* larvae with *B. burgdorferi* (*s.l.*) (${RI}_{bb_i}$).

As our study contained animals that were collected from a range of habitat types and we wanted to model a host community in a forested habitat, we estimated *I. ricinus* burdens for the four species of mustelid described in this study by modelling tick burden as a function of forest cover. First, we used the Pan-European forest map for 2000 [46] and estimated the percentage forest cover within a buffer with a 564.2 m radius (~1 km^2^: the average home range size of the studied species [23]). We did this for a subset of the roadkill samples with information on the exact location where the animal was found and used the map of 2000 as this year overlapped with both the sampling in the Netherlands and Belgium. Secondly, we modelled individual *I. ricinus* burden as a function of species and percentage forest cover using a generalized linear model (GLM) with a negative binomial distribution and a log link function and used the model fit to predict the tick burden for each of the four species in a site with 100% forest cover. We combined estimates of female and male *I. ricinus* to estimate the adult *I. ricinus* burden. In addition to these predicted tick burdens, we used average densities for badger, polecat and pine marten as published in the PanTHERIA database [47]. Stone marten density was not available from PanTHERIA and there are, to our knowledge, no recent estimates of average stone marten densities in forested habitats. We therefore used the same estimate as for the related pine marten.

### Statistical analyses

We tested for differences between the species and the two countries in which animals were collected assuming that biases due to habitat and season were similar between species and countries. We did this to be able to use the full dataset, as only a subset contained full information on location and date when the animal was collected. We used GLMs with a negative binomial distribution and a log link function to test for differences in tick burden between the different mustelid species while accounting for overdispersion in the data. We also took into account potential differences between the two countries as a factor, resulting in GLMs with two factors as independent variables: species and country. Each tick species and life stage was tested separately except for males of both tick species, due to inadequate sample size. Similarly, we used GLMs with a binomial distribution and a log link function to test for differences in infection rate between the different mustelid species. Again, we included country as a factor in all models, resulting in GLMs with two factors as independent variables, species and country, and ran the same model for each pathogen. We defined animals as being infected when at least one tissue sample was found positive for the pathogen. All models were run in R 3.4.4. We performed a Tukey HSD *post-hoc* test to test for differences between the species using the *multcomp* package [48].

In a second part of the analysis, we used GLMs with a binomial distribution and a logit link function to test for differences in infection rate for the different pathogens in feeding ticks. First, we tested for a difference between feeding nymphs from the combined pool of mustelids and questing nymphs, for which we used data on the infection rate of questing *I. ricinus* nymphs from three recent studies in Belgium [49–51] and one from the Netherlands [52]. We performed this analysis to test for a potential increase in infection rate in feeding nymphs, as an indication that the mustelid species might be able to transmit the different pathogens. Secondly, we tested for differences between feeding nymphs and feeding adults from the combined pool of mustelids, again as an indication that the mustelid species might be able to transmit the different pathogens.

## Results

### Tick burden

In total, 637 animals were inspected for the presence of ticks. Only half of the animals carried ticks (*n* = 308), and less than 20% of the animals carried ~80% of the ticks (Table 1). We collected a total of 642 *I. ricinus* and 2621 *I. hexagonus* and did not find any other ixodid species, such as *Ixodes rugicollis* or *I. canisuga*. In general, pine martens had the highest *I. ricinus* burden (Table 2 and Additional file 1: Table S1). For larvae, pine martens had a higher burden than badgers (*β* = 4.7, *Z* = 3.2, *P* = 0.006) and stone martens (*β* = 4.1, *Z* = 4.7, *P* < 0.0001). For nymphs, pine martens had a higher burden than stone martens (*β* = 2.7, *Z* = 3.5, *P* = 0.003). For females, stone martens had a higher burden than polecats (*β* = 1.2, *Z* = 2.6, *P* = 0.049). We found a consistent difference between the two countries, where mustelids carried more larvae (*β* = 4.0, *Z* = 2.3, *P* = 0.02), nymphs (*β* = 5.4, *Z* = 4.1, *P* < 0.0001) and females (*β* = 2.4, *Z* = 5.3, *P* < 0.0001) in the Netherlands compared to Belgium.

**Table 1:** Tick burden of the different mustelid species

Species	*Meles meles*		*Mustela putorius*		*Martes martes*		*Martes foina*	
Animals screened for ticks (*n*)	123		385		53		76	
Animals without ticks (*n*, %)	84	68%	201	52%	8	15%	36	47%
Animals with 80% of ticks (*n*, %)	15	12%	58	15%	10	19%	14	18%

**Table 2:** Tick burden of the different stages of *I. ricinus* and *I. hexagonus*. Tick burden is expressed as the mean number of ticks per host individual. Superscript letters indicate significant differences between species (*P* < 0.05), where estimates increase from a-c, as described in Methods

Species	*Meles meles*		*Mustela putorius*		*Martes martes*		*Martes foina*	
Tick species/stage	Total	Mean	Total	Mean	Total	Mean	Total	Mean
*I. ricinus* larvae	1	0.01^a^	1	0.00^ab^	366	6.91^b^	4	0.05^a^
*I. ricinus* nymphs	2	0.02^ab^	6	0.02^ab^	67	1.26^b^	3	0.04^a^
*I. ricinus* males	0	0.00	0	0.00	9	0.17	11	0.14
*I. ricinus* females	16	0.13^ab^	19	0.05^a^	87	1.64^ab^	50	0.66^b^
*I. hexagonus* larvae	4	0.03^a^	761	1.98^c^	507	9.57^bc^	75	0.99^ab^
*I. hexagonus* nymphs	105	0.85^a^	607	1.58^b^	90	1.70^a^	69	0.91^a^
*I. hexagonus* males	0	0.00	18	0.05	1	0.02	1	0.01
*I. hexagonus* females	43	0.35^b^	315	0.82^c^	11	0.21^a^	14	0.18^a^

*Ixodes hexagonus* showed a different pattern, where polecats generally had the highest burden (Table 2 and Additional file 1: Table S1). For larvae, polecats had a higher burden than badgers (*β* = 4.5, *Z* = 4.9, *P* < 0.0001) and stone martens (*β* = 2.4, *Z* = 3.3, *P* = 0.005). For nymphs, polecats had a higher burden than badgers (*β* = 1.1, *Z* = 3.8, *P* < 0.0001), pine martens (*β* = 2.3, *Z* = 4.0, *P* < 0.0001) and stone martens (*β* = 2.2, *Z* = 4.8, *P* < 0.0001). For females, polecats had a higher burden than badgers (*β* = 1.1, *Z* = 3.3, *P* = 0.004), pine martens (*β* = 2.9, *Z* = 4.0, *P* < 0.001) and stone martens (*β* = 2.6, *Z* = 4.5, *P* < 0.001), and badgers had a higher burden than pine martens (*β* = 1.8, *Z* = 2.6, *P* = 0.04) and stone martens (*β* = 1.5, *Z* = 2.6, *P* = 0.04). Again, we found a consistent difference between the two countries, where mustelids carried more larvae (*β* = 3.9, *Z* = 5.2, *P* < 0.0001), nymphs (*β* = 2.6, *Z* = 5.7, *P* < 0.0001) and females (*β* = 1.5, *Z* = 2.8, *P* = 0.005) in the Netherlands compared to Belgium.

### Infection rate in animals

In total, organs of 789 animals were inspected for pathogens. Based on the qPCR analyses of spleen, liver and ear samples, we detected infection with *A. phagocytophilum* in 27 polecats (*n* = 556), 11 pine martens (*n* = 51), two badgers (*n* = 114) and one stone marten (*n* = 68) (Table 3). There were no differences in infection rate between the species (Additional file 1: Table S2), but infection rate was higher in the animals from the Netherlands compared to Belgium (odds ratio: 7.2, *Z* = 2.7, *P* = 0.006). Based on conventional PCR followed by sequencing on the *Anaplasma*-positive samples, liver samples from three polecats could be typed further as ecotype I [44]. DNA of *B. burgdorferi* (*s.l.*) was found in organs of two stone martens, two pine martens, one polecat and one badger. Spleen, liver and ear samples were found positive for *B. burgdorferi* (*s.l.*). Again, there was no difference in infection rate between the species (Additional file 1: Table S2), but the infection rate was higher in the samples from the Netherlands compared to Belgium (odds ratio: 44.9, *Z* = 2.3, *P* = 0.02). Despite several attempts, only two *B. burgdorferi* (*s.l.*)*-*positive samples could be typed to the genospecies level. *Borrelia afzelii* was identified in two organs from two different stone martens (Table 3). Two pine martens, one stone marten and one polecat were positive for *N. mikurensis*, whereas we did not detect *B. miyamotoi* DNA in any of the 789 animals tested. DNA of *N. mikurensis* was detected in spleen, liver and ear samples. There was no difference in infection rate for *N. mikurensis* between the species (Additional file 1: Table S2) or between the countries (odds ratio: 14.4, *Z* = 1.1 *P* = 0.27).

**Table 3:** Presence of tick-borne pathogens in mustelid tissues. DNA lysates from mustelid liver (L), spleen (S) and ear (E) biopsies were tested by qPCR for tick-borne pathogens. Number of positive animals and infection rates of each pathogen are shown

Species	*Meles meles*	*Mustela putorius*	*Martes martes*	*Martes foina*
Common name	European badger	European polecat	Pine marten	Stone marten
Animals (*n*)	114	556	51	68
Liver, spleen, ear (*n*)	113 (L), 11 (S), 4 (E)	556 (L), 4 (S)	50 (L), 50 (S), 26 (E)	67 (L), 26 (S)^a^, 21 (E)
*B. burgdorferi* (*s.l.*) (*n*: %)	1: 0.9% (L)	1: 0.2% (L)	2: 3.9% (S)	2: 2.9% (E)
*B. miyamotoi* (*n*: %)	0: 0.0%	0: 0.0%	0: 0.0%	0: 0.0%
*N. mikurensis* (*n*: %)	0: 0.0%	1: 0.2% (L)	2: 3.9% (S, E)	1: 1.5% (L)
*A. phagocytophilum* (*n*: %)	2: 1.8% (L, S)	27: 4.9% (L)^b^	11: 22% (L, S, E)	1: 1.5% (L, S, E)

^a^Two samples were identified as *B. afzelii* by conventional PCR followed by sequencing

^b^Isolates from three individuals were identified as ecotype I by conventional PCR followed by sequencing. All other *Anaplasma* and *Borrelia*-positive samples could not be typed further

### Infection rate in feeding ticks

In total, 1828 feeding ticks, both *I. ricinus* (*n* = 215) and *I. hexagonus* (*n* = 1613), were removed from the mustelids and tested for the presence of four tick-borne pathogens (Table 4). From the 15 *I. ricinus* larvae feeding on pine martens, only one tested positive for *B. miyamotoi* DNA, and all tested negative for *B. burgdorferi* (*s.l.*), *N. mikurensis* and *A. phagocytophilum.* The two feeding larvae from a polecat (*n* = 1) and a stone marten (*n* = 1) were negative for all four pathogens (Table 4). All four pathogens were found in feeding *I. ricinus* nymphs and adults, with nymphal infection rates of 7.5% [*B. burgdorferi* (*s.l.*)], 2.5% (*B. miyamotoi*), 23% (*N. mikurensis*) and 10% (*A. phagocytophilum*). We compared these to infection rates of questing *I. ricinus* nymphs (*n* = 10,628) based on previous studies [41, 42], which were 13.3% for *B. burgdorferi* (*s.l.*), 2.5% for *B. miyamotoi*, 3.7% for *N. mikurensis* and 4.0% for *A. phagocytophilum.* The infection rate with *N. mikurensis* was higher in feeding *I. ricinus* nymphs collected from mustelids compared to questing nymphs (odds ratio = 7.6, *Z* = 5.3, *P* < 0.0001), but not for *B. burgdorferi* (*s.l.*) (odds ratio = 0.53, *Z* = 1.1, *P* = 0.29), *B. miyamotoi* (odds ratio = 1.0, *Z* = 0.0, *P* = 1.00) or *A. phagocytophilum* (odds ratio = 2.7, *Z* = 1.9, *P* = 0.06). We found adult *I. ricinus* infection rates of 13% [*B. burgdorferi* (*s.l.*)], 3.8% (*B. miyamotoi*), 6.3% (*N. mikurensis*) and 21% (*A. phagocytophilum*). The infection rate was lower in feeding *I. ricinus* adults compared to nymphs for *N. mikurensis* (odds ratio = 4.3, *Z* = 2.9, *P* = 0.004), but not for the other pathogens (Additional file 1: Table S3).

**Table 4:** Presence of tick-borne pathogens in ticks feeding on mustelids. DNA lysates from *I. ricinus* (*I. ric*) and *I. hexagonus* (*I. hex*) feeding on mustelids (*n*) were tested by qPCR for tick-borne pathogens. Number of pathogen-positive ticks are shown, as well as the number of animals with positive ticks. Infection rate is only shown when more than 10 ticks were tested

	*Larvae* (*n*)		*Nymphs* (*n*)		*Adults* (*n*)		Animals (*n*)	
	*I.ric*	*I.hex*	*I.ric*	*I.hex*	*I.ric*	*I.hex*	*I.ric*	*I.hex*
*Meles meles* (*n* = 38)	(*n* = 0)	(*n* = 2)	(*n* = 1)	(*n* = 27)	(*n* = 11)	(*n* = 25)	(*n* = 5)	(*n* = 37)
*B. burgdorferi* (*s.l.*) (%)	–	0	0	0 (0)	5 (45)	1 (4)	3	2 (5)
*B. miyamotoi* (%)	–	0	0	0 (0)	0 (0)	0 (0)	0	0 (0)
*N. mikurensis* (%)	–	0	0	0 (0)	0 (0)	0 (0)	0	0 (0)
*A. phagocytophilum* (%)	–	0	0	1 (4)	0 (0)	1 (4)	0	2 (5)
*Mustela putorius* (*n* = 364)	(*n* = 1)	(*n* = 204)	(*n* = 9)	(*n* = 742)	(*n* = 46)	(*n* = 490)	(*n* = 43)	(*n* = 358)
*B. burgdorferi* (*s.l.*) (%)	0	0 (0)	0	6 (1)	4 (9)	11 (2)	4 (9)	15 (4)
*B. miyamotoi* (%)	0	0 (0)	0	0 (0)	1 (3)	0 (0)	1 (2)	0 (0)
*N. mikurensis* (%)	0	0 (0)	2	1 (0)	0 (0)	0 (0)	0 (0)	1 (0)
*A. phagocytophilum* (%)	0	2 (1)	1	30 (4)	7 (15)	47 (10)	7 (16)	50 (14)
*Martes martes* (*n* = 44)	(*n* = 15)	(*n* = 22)	(*n* = 27)	(*n* = 36)	(*n* = 57)	(*n* = 9)	(*n* = 33)	(*n* = 26)
*B. burgdorferi* (*s.l.*) (%)	0 (0)	0 (0)	2 (7)	0 (0)	4 (7)	0	6 (18)	0 (0)
*B. miyamotoi* (%)	1 (15)	0 (0)	1 (4)	0 (0)	3 (5)	0	4 (12)	0 (0)
*N. mikurensis* (%)	0 (0)	0 (0)	6 (22)	2 (6)	5 (9)	3	8 (24)	3 (12)
*A. phagocytophilum* (%)	0 (0)	0 (0)	3 (11)	5 (14)	24 (42)	4	15 (45)	5 (19)
*Martes foina* (*n* = 39)	(*n* = 1)	(*n* = 16)	(*n* = 3)	(*n* = 31)	(*n* = 44)	(*n* = 9)	(*n* = 22)	(*n* = 24)
*B. burgdorferi* (*s.l.*) (%)	0	0 (0)	1	0 (0)	8 (18)	0	5 (23)	0 (0)
*B. miyamotoi* (%)	0	0 (0)	0	0 (0)	2 (5)	0	1 (5)	0 (0)
*N. mikurensis* (%)	0	1 (6)	1	2 (6)	5 (11)	1	6 (27)	4 (17)
*A. phagocytophilum* (%)	0	0 (0)	0	0 (0)	2 (5)	0	2 (9)	0 (0)

None of the 1613 *I. hexagonus* ticks feeding on mustelids tested positive for *B. miyamotoi* (Table 4). Two *I. hexagonus* larvae from polecats tested positive for *A. phagocytophilum* (1%). One larvae from a stone marten tested positive for *N. mikurensis* (6%). The three other pathogens were found in feeding *I. hexagonus* nymphs (*n* = 836) and adults (*n* = 533), with nymphal infection rates of 0.7% [*B. burgdorferi* (*s.l.*)], 0.6% (*N. mikurensis*) and 4.3% (*A. phagocytophilum*). The infection rates in *I. hexagonus* adults were higher than in *I. hexagonus* nymphs for *B. burgdorferi* (*s.l.*) (2.3%: odds ratio = 3.2, *Z* = 2.3, *P* = 0.02), and *A. phagocytophilum* (9.8%: odds ratio = 2.4, *Z* = 3.9, *P* < 0.0001), but not for *N. mikurensis* (0.8%: odds ratio = 1.3, *Z* = 0.3, *P* = 0.73; Additional file 1: Table S3). The infection rates in *I. hexagonus* nymphs were lower than the infection rates in *I. ricinus* nymphs for *B. burgdorferi* (*s.l.*) (odds ratio = 11.2, *Z* = 3.3, *P* = 0.0009) and *N. mikurensis* (odds ratio = 48.3, *Z* = 6.6, *P* < 0.0001), but not for *A. phagocytophilum* (odds ratio = 2.5, *Z* = 1.6, *P* = 0.10; Additional file 1: Table S3). Infection rates in *I. hexagonus* adults were consistently lower than infection rates in *I. ricinus* adults (*B. burgdorferi* (*s.l.*): odds ratio = 6.7, *Z* = 5.1, *P* < 0.0001; *A. phagocytophilum*: odds ratio = 2.4, *Z* = 3.7, *P* = 0.0003; *N. mikurensis*: odds ratio = 8.9, *Z* = 3.7, *P* = 0.0003).

### Relative importance of mustelids in maintaining *I. ricinus* and *B. burgdorferi* (*s.l.*)

Because we found few *I. ricinus* larvae feeding on any of the mustelid species, the realized reservoir competences of these hosts could not be determined. Therefore, only the relative importance of the four host species in terms of feeding the different stages of *I. ricinus* was determined (Table 5). Modelled *I. ricinus* burdens were based on a subset of 110 badgers, 52 pine martens, 307 polecats and 69 stone martens for which we could estimate the percentage forest cover around the location where they were found. This percentage did not differ between species or countries (GLM with binomial distribution and logit link, all *P* > 0.2). In this subset, *I. ricinus* larval burden increased with forest cover (*β* = 3.7, *Z* = 3.1, *P* = 0.002), while nymphal burden (*β* = -0.4, *Z* = -0.4, *P* = 0.70) and female burden (*β* = 1.0, *Z* = 1.5, *P* = 0.13) did not. Using fitted GLMs to predict *I. ricinus* burden at 100% forest cover for each of the mustelid species resulted in the estimated tick burdens in Table 5. Due to their relatively low burden and low density, estimated relative importance for all four mustelid species was low, with the highest estimate of 0.5% of the adult *I. ricinus* fed by pine martens.

**Table 5:** Relative importance of mustelids for feeding the different stages of *I. ricinus* and for infecting *I. ricinus* larvae with *B. burgdorferi* (*s.l.*). Vertebrate species considered in our model host-community and the density that was used in these calculations. Mustelid species are shown in bold

Species	Density (1 km^-2^)	Average *I. ricinus* burden			Realized reservoir competence	Relative importance for feeding/infecting (%)			
		Larvae	Nymphs	Adults		Larvae	Nymphs	Adults	*B. burgdorferi* (*s.l.*)
*Apodemus sylvaticus*	1200	5.2	0.1	0.0	0.2	31.8	5.7	0.0	15.8
*Microtus agrestis*	1000	5.6	0.4	0.0	0.6	28.6	14.8	1.2	51.6
*Myodes glareolus*	1200	4.7	0.2	0.0	0.3	28.7	7.0	0.0	21.8
*Erinaceus europaeus*	1	119.9	58.7	10.5	na	0.6	2.2	3.4	na
*Capreolus capreolus*	11	20.4	18.5	25.3	na	1.1	7.6	91.0	na
*Vulpes vulpes*	1	0.0	1.3	4.2	na	0.0	0.0	1.4	na
***Meles meles***	**2.52**	**0.1**	**0.0**	**0.3**	**na**	**0.0**	**0.0**	**0.3**	**na**
***Mustela putorius***	**0.84**	**0.1**	**0.0**	**0.1**	**na**	**0.0**	**0.0**	**0.0**	**na**
***Martes martes***	**0.44**	**47.5**	**1.0**	**3.6**	**na**	**0.1**	**0.0**	**0.5**	**na**
***Martes foina***	**0.44**	**1.5**	**0.0**	**1.9**	**na**	**0.0**	**0.0**	**0.3**	**na**
*Cyanistes caeruleus*	200	0.1	0.0	0.0	na	0.1	0.1	0.0	na
*Erithacus rubecula*	80	1.5	0.3	0.0	0.0	0.6	0.9	0.0	0.1
*Fringilla coelebs*	100	1.2	0.1	0.0	na	0.6	0.5	0.0	na
*Parus major*	100	0.8	0.3	0.0	0.2	0.4	1.0	0.0	0.2
*Phylloscopus collybita*	100	0.3	0.1	0.0	na	0.1	0.3	0.0	na
*Prunella modularis*	200	2.2	2.6	0.0	0.0	2.3	19.4	0.0	0.3
*Sylvia atricapilla*	40	0.5	0.2	0.0	0.0	0.1	0.3	0.0	0.0
*Turdus merula*	200	3.6	4.3	0.0	0.8	3.6	32.5	1.4	8.5
*Turdus philomelos*	80	2.8	2.6	0.0	0.5	1.2	7.6	0.5	1.8

The realized reservoir competence is the proportion of feeding larvae that are infected with *B. burgdorferi* (*s.l.*). In these calculations, the relative importance for feeding ticks is dependent on the vertebrate composition, the densities of vertebrate species and their tick burdens (see Equation 1 and Hofmeester *et al.* [18]).

*Abbreviation*: na, not applicable

## Discussion

Unravelling which host species maintain tick and pathogen populations is important for understanding pathogen dynamics and for developing measures to reduce disease burden [1]. In this study, we investigated the tick burden and infection rate with four tick-borne pathogens of four species of mustelid (badger, pine marten, polecat and stone marten) that generally occur in European forests [24]. Overall, we found relatively low *I. ricinus* and *I. hexagonus* burdens on all four mustelid species and low infection rates with three pathogens *Anaplasma phagocytophilum*, *Borrelia burgdorferi* (*s.l.*) and *Neoehrlichia mikurensis*. Given the low densities in which these four host species generally occur, this leads us to conclude that these common mustelids play a limited role in maintaining *I. ricinus* populations and in infecting *I. ricinus* with tick-borne pathogens in forested areas.

The distribution of *I. ricinus* on mustelids was highly overdispersed, like many other vertebrate species [53], and summarizing this distribution by a mean value might not result in the most accurate parameter [54]. However, we still chose to present the mean (Table 2) to make our findings comparable with other studies. Subsequently, we did take overdispersion into account in our analyses of tick burden. Pine martens had the highest *I. ricinus* burden for all stages. This could be due to the fact that pine martens were only collected in the Netherlands and mustelids in the Netherlands had a higher *I. ricinus* burden. However, it is more likely due to the habitat preference of both tick and host. *Ixodes ricinus* is mainly found in forested areas [18, 55–58], which is also the habitat that is preferred by the pine marten [59]. This was further supported by a positive correlation of *I. ricinus* burden with the percentage forest cover around the roadkill site for some tick stages. The three other mustelid species are regularly found in forested areas, but also spend a considerable amount of time in other, more open, habitats [23], which could explain their lower *I. ricinus* burden.

As only a few previous studies have been performed on the burden of *I. ricinus* on mustelids, it is difficult to put our results into perspective. We found a higher prevalence of infestation and average *I. ricinus* burden on pine martens, stone martens and badgers than in previous studies [18, 25, 26, 60]. Two other studies [22, 23] used a similar methodology, using road-killed animals. The risk with using road-killed animals is that ticks might have left the host before collection. We tried to collect animals as soon as possible after they died, but the exact timing of death could not be determined. Therefore, our estimates of tick burden might be underestimations due to loss of ticks *post-mortem*. However, as Christian [60] found similar tick burdens on live-trapped animals, we think our estimates are representative. Furthermore, several studies on other host species also used road-killed specimens, increasing the comparability between datasets [25, 61].

We found a consistently lower number of *I. ricinus* of all stages on badgers, polecats and stone martens collected in Belgium compared to the Netherlands. This could be caused by a difference in sampling between the two countries. For example, almost all Belgian (i.e. Flemish) animals checked for ticks were collected before 2006, while most Dutch specimens were collected and screened after 2006 and up to 2015. Therefore, the difference in tick burden might be caused by spatio-temporal variations in abundance of ticks and of their infection rate in the past decades [62]. Another explanation might be a difference between the two countries in habitat where the mustelids were collected. This is, however, unlikely as there was no difference in estimated forest cover around the road-kill site between species or countries. There might have been a difference in forest composition between the countries, which could result in differences in questing tick densities and infection rate [63]. A more elaborate analysis, including detailed information on the landscape surrounding the place where the animals were found, is needed to further investigate this relationship.

All four mustelid species were infested with *I. hexagonus* of all stages, suggesting that they play an important role in maintaining *I. hexagonus* populations. Little is known about the population dynamics of *I. hexagonus*, although previous studies have described the tick species to be parasitizing several members of the *Mustelidae* family: the common weasel, stoat (*Mustela erminea*), polecat, badger and otter [27, 29]. Furthermore, hedgehogs (*Erinaceus europaeus* and *Erinaceus roumanicus*) have often been found to host *I. hexagonus* [34, 35]. As *I. hexagonus* is a nidiculous tick species (searching for a host in the nest or burrow) and as all these vertebrate species use different burrows to nest in, it is possible that *I. hexagonus* forms many cryptic subpopulations with only occasional genetic exchange between subpopulations, potentially giving rise to host races as was found for *I. uriae* [64]. Based on our results, it seems possible that polecats, and to some extent all the studied mustelids, can play a role in maintaining *I. hexagonus* subpopulations.

*Borrelia burgdorferi* (*s.l.*) and *A. phagocytophilum* DNA were detected in tissue samples of European badgers, polecats, pine martens and stone martens (Table 3), suggesting that all four mustelid species can be infected and consequently act as reservoir hosts for these two pathogens. *Neoehrlichia mikurensis* DNA was present in organs of polecats, stone martens and pine martens, but not in badgers. One explanation might be that badgers are not a reservoir host for *N. mikurensis*. Recent studies provide evidence that badgers and foxes are reservoir hosts for a related *Neoehrlichia* species [65]. *Borrelia miyamotoi* was not detected in any of the 867 investigated organ samples (Table 3), indicating that these animals do not play a major role in the transmission of *B. miyamotoi*. The higher infection rate in mustelids collected in the Netherlands compared to the animals collected in Belgium could simply be a response to the difference in tick burden we found between the countries as the two parameters are highly correlated [18].

This study assessed, by means of molecular methods, the circulation of four pathogens in their enzootic cycle. The presence of the DNA of a pathogen in internal organs such as liver or spleen is an indication that the host is infected [66, 67]. Although culturing is considered the most reliable method in determining the presence of viable or infectious microorganisms, it suffers from low sensitivity. Furthermore, it is costly, time consuming and difficult, if not impossible for some pathogens [3]. Our investigation only tested for the presence of DNA from four pathogens and not their viability or infectivity. However, previous studies implicate *I. ricinus* as their vector and the investigated animals as potential, but poorly studied, hosts [35, 68, 69]. Therefore, the inability of a DNA-based detection method to assess infectiousness in host species was expected to be a minor issue. On the other hand, the absence of detectable DNA from these microorganisms in tick lysates or animal tissues does not imply complete absence of these infectious agents either as pathogens might be present in tissues in concentrations lower than the detection limit of the PCR-array. Furthermore, some pathogens might be missed. For example *B. miyamotoi*, might give rise to short-term, limited infections [70, 71], whereas the tissue tropism for *B. afzelii* is most likely skin, rather than internal organs [72, 73]. As a result, one should be careful when interpreting results based on only PCR methods as in this study.

Although we detected *B. burgdorferi* (*s.l.*) DNA in organs of all mustelid species, we did not find any feeding larvae that were infected with *B. burgdorferi* (*s.l.*). Only one study previously investigated the infection rate of *B. burgdorferi* (*s.l.*) in organs for any of the species in this study. Gern & Sell [69] found a far higher infection rate compared to our findings. This could be due to the larger number of skin samples tested by these authors, which is the preferred tissue for *B. afzelii*. The high infection rate in their study could also, however, have been a result of the low sample size (6 animals). Infection rate in feeding *I. ricinus* nymphs was similar to that in questing nymphs. These results suggested that none of the studied animal species are important reservoirs for *B. burgdorferi* (*s.l.*) Unfortunately, the number of feeding larvae that we found on the mustelids was too low to be able to calculate a representative realized reservoir competence [18].

The infection rate of *B. burgdorferi* (*s.l.*), *A. phagocytophilum* and *N. mikurensis* were higher in feeding *I. hexagonus* adults compared to feeding *I. hexagonus* nymphs, a pattern that would be expected when a host species amplifies a pathogen [35, 44]. Furthermore, two *I. hexagonus* larvae feeding on polecats were infected with *A. phagocytophilum*, while one *I. hexagonus* larva feeding on a stone marten was infected with *N. mikurensis.* However, these pathogens are not transovarially transmitted [74]. Therefore, these findings suggest that the studied mustelids can transmit these pathogens to *I. hexagonus* ticks. In theory, *I. hexagonus* can function as a bridging vector for some tick-borne pathogens, maintaining enzootic cycles with occasional spillover to *I. ricinus*. However, the low densities of mustelids and relatively low *I. ricinus* burden plus the low number of people that are bitten by *I. hexagonus* [30, 75] indicate that this enzootic cycle does not pose a public health risk. The absence of *B. miyamotoi* DNA in the investigated *I. hexagonus* (*n* = 1613), and tissues from mustelids (*n* = 789) indicate that this tick species and these animal species do not play a significant role in the enzootic transmission cycle of *B. miyamotoi*.

Given the relatively low densities of badgers, pine martens, polecats and stone martens in forest areas (0.44–2.52 km^-2^), and the low to moderate abundance of *I. ricinus* feeding on them (larvae: 0.1–47.5 individual^-1^, nymphs: 0–1 individual^-1^, adults: 0.1–3.6 individual^-1^; Table 5), their relative importance in feeding any stage of *I. ricinus* is low. Species with a high relative importance for maintaining the different stages of *I. ricinus* all have either high average *I. ricinus* burdens (roe deer: 25.3 adults individual^-1^), high density (small rodents: 1000–1200 km^-2^) or moderate *I. ricinus* burden and density (blackbird *Turdus merula*: 4.3 nymphs individual^-1^ and 200 km^-2^) [18]. Although we could not quantify the realized reservoir competence for *B. burgdorferi* for any of the mustelid species, we infer that the relative importance of mustelids in the transmission cycle of *B. burgdorferi* (*s.l.*), and other tick-borne pathogens, is low due to their low population density, low *I. ricinus* burden and low infection rate with pathogens (0.2–3.9%). Hofmeester et al. [18] showed that transmission cycles can exist when hosts reach either high densities (small rodents) or high infection rates (blackbirds: 86%), neither of which is the case for the four species of mustelid. Quantitative (meta) analyses on the relative importance of vertebrate hosts in the transmission cycle of *B. miyamotoi*, *A. phagocytophilum* and *N. mikurensis* have never been performed and are highly needed to understand which animals contribute most to the disease risk of these tick-borne pathogens [1, 63]. As all four mustelid species occur in (peri-)urban areas, they might act as propagation hosts for adult *I. ricinus* [76, 77], and might be involved in maintaining relatively small populations of *I. ricinus* in these areas*.*

## Conclusions

Although all stages of *I. ricinus* were found on mustelids, the relative contribution of mustelids to the life-cycle of this tick species in forested areas is low due to low host densities and tick burdens. Consequently, we conclude that their relative contribution to the enzootic cycle of tick-borne pathogens is low. Moreover, a recent study showed that (meso-)carnivores, such as foxes and stone martens, can lower the number of ticks feeding on rodents, and therefore may have cascading negative effects on tick-borne disease risk [78]. Therefore, the role of mustelids in *I. ricinus*-borne pathogen dynamics is more likely indirect through species interactions with their prey than direct as hosts for ticks and pathogens. In contrast, all four mustelid species carried all stages of *I. hexagonus*, potentially maintaining an enzootic cycle of this species, apart from the cycle involving hedgehogs as main host species. Similarly, as badgers, martens and polecats carry adult *I. ricinus*, they might be able to act as propagation hosts in sites where deer are absent [56]. As such, they may contribute to the maintenance of *I. ricinus* populations in (peri)-urban areas. Finally, our study shows the importance of filling knowledge gaps in terms of the tick burden and infection rate of little-studied host species that occur in many European forests [1, 18].

## Additional file

Additional file 1: **Table S1.** Estimated differences from outcomes of models. Model outcomes of generalized linear models with a negative binomial distribution and log link function testing for differences in tick burden between the mustelid species while correcting for differences between countries. One model per tick species and life stage combination. Estimated differences in tick burden (on a log scale) and standard error (between brackets) are given for each combination of species. The species in the first column is the “base” value, and *P*-values, based on a Tukey *post-hoc* test, are represented by · for *P* < 0.1, * for *P* < 0.05, ** for *P* < 0.01 and *** for *P* < 0.001. **Table S2.** Estimated odds ratio from outcomes of models. Model outcomes of generalized linear models with a binomial distribution and logit link function testing for differences in infection prevalence between the mustelid species while correcting for differences between countries. One model per microorganism. Estimated ln(odds ratio) and standard error are given for each combination of species. The species in the first column is the “base” value. *P*-values based on a Tukey *post-hoc* test were all > 0.1 and are not presented in the table. **Table S3.** Estimated odds ratio from outcomes models. Model outcomes of generalized linear models with a binomial distribution and logit link function testing for differences in infection prevalence between feeding ticks of different stages found on all mustelids. Each cell gives the outcome of a single model. Estimated ln(odds ratio) and standard error are given for each combination. *P*-values are represented by **·** for *P* < 0.1, * for *P* < 0.05, ** for *P* < 0.01 and *** for *P* < 0.001. **Table S4.** DNA sequences of *B. afzelii* (IGS) and *A. phagocytophilum* (GroEL) from tissue samples. (DOCX 28 kb)

## Abbreviations

C_q_: Quantification cycle
GLM: Generalized linear models
IGS: 5S-23S intergenic spacer region
LB: Lyme borreliosis
TBP: tick-borne pathogens
