# Supplementary material for: Role of mustelids in the life-cycle of ixodid ticks and transmission cycles of four tick-borne pathogens
Source: Parasit Vectors. 2018 Nov 20;11:600. doi: 10.1186/s13071-018-3126-8 (PMC6245527; doi:10.1186/s13071-018-3126-8)
Supplement: Supplementary file 1 — Table S1. Estimated differences from outcomes of models. Model outcomes of generalized linear models with a negative binomial distribution and log link function testing for differences in tick burden between the mustelid species while correcting for differences between countries. One model per tick species and life stage combination. Estimated differences in tick burden (on a log scale) and standard error (between brackets) are given for each combination of species. The species in the first column is the “base” value, and P-values, based on a Tukey post-hoc test, are represented by · for P < 0.1, * for P < 0.05, ** for P < 0.01 and *** for P < 0.001. Table S2. Estimated odds ratio from outcomes of models. Model outcomes of generalized linear models with a binomial distribution and logit link function testing for differences in infection prevalence between the mustelid species while correcting for differences between countries. One model per microorganism. Estimated ln(odds ratio) and standard error are given for each combination of species. The species in the first column is the “base” value. P-values based on a Tukey post-hoc test were all > 0.1 and are not presented in the table. Table S3. Estimated odds ratio from outcomes models. Model outcomes of generalized linear models with a binomial distribution and logit link function testing for differences in infection prevalence between feeding ticks of different stages found on all mustelids. Each cell gives the outcome of a single model. Estimated ln(odds ratio) and standard error are given for each combination. P-values are represented by · for P < 0.1, * for P < 0.05, ** for P < 0.01 and *** for P < 0.001. Table S4. DNA sequences of B. afzelii (IGS) and A. phagocytophilum (GroEL) from tissue samples. (DOCX 28 kb) [file 13071_2018_3126_MOESM1_ESM.docx]

**Additional file 1: Table S1.** Estimated differences from outcomes of generalized linear models. Model outcomes of generalized linear models with a negative binomial distribution and log link function testing for differences in tick burden between the four mustelid species while correcting for differences between countries. One model per tick species and life stage combination. Estimated differences in tick burden (on a log scale) and standard error (between brackets) are given for each combination of species. The species in the first column is the “base” value, and *P*-values, based on a Tukey *post-hoc* test, are represented by · for *P* < 0.1, * for *P* < 0.05, ** for *P* < 0.01 and *** for *P* < 0.001.

|  | Pine marten | Polecat | Stone marten |
| --- | --- | --- | --- |
| ***Anaplasma phagocytophilum*** |  |  |  |
| Badger | 1.2 (0.9) | 1.5 (0.8) **·** | 0.8 (0.9) |
| Pine marten | - | 0.3 (0.8) | -0.5 (0.6) |
| Polecat | - | - | -0.7 (0.8) |
| Stone marten | - | - | - |
| ***Borrelia burgdorferi* s.l.** |  |  |  |
| Badger | -0.6 (1.3) | -0.2 (1.7) | -0.0 (1.3) |
| Pine marten | - | 0.4 (1.8) | 0.6 (1.0) |
| Polecat | - | - | 0.2 (1.7) |
| Stone marten | - | - | - |
| ***Neoehrlichia mikurensis*** |  |  |  |
| Badger | 20.8 (2E4) | 20.2 (2E4) | 20.5 (2E4) |
| Pine marten | - | -0.5 (2.5) | -0.2 (1.3) |
| Polecat | - | - | 0.3 (2.5) |
| Stone marten | - | - | - |

**Additional file 1: Table S2.** Estimated odds ratio from outcomes of generalized linear models. Model outcomes of generalized linear models with a binomial distribution and logit link function testing for differences in infection prevalence between the four mustelid species while correcting for differences between countries. One model per microorganism. Estimated ln(odds ratio) and standard error (between brackets) are given for each combination of species. The species in the first column is the “base” value. *P*-values based on a Tukey *post-hoc* test were all > 0.1 and are not presented in the table.

|  | Pine marten | Polecat | Stone marten |
| --- | --- | --- | --- |
| ***Ixodes ricinus* larvae** |  |  |  |
| Badger | 4.7 (1.4)** | 0.6 (1.4) | 0.5 (1.5) |
| Pine marten | - | -4.1 (1.8)* | -4.1 (0.9)*** |
| Polecat | - | - | -0.1 (1.8) |
| Stone marten | - | - | - |
| ***Ixodes ricinus* nymphs** |  |  |  |
| Badger | 2.1 (1.0)* | 2.0 (1.5) | -0.6 (1.2) |
| Pine marten | - | -0.1 (1.2) | -2.7 (0.8)*** |
| Polecat | - | - | -2.6 (1.4) **·** |
| Stone marten | - | - | - |
| ***Ixodes ricinus* females** |  |  |  |
| Badger | 1.2 (0.6)* | 0.1 (0.5) | 1.3 (0.5)* |
| Pine marten | - | -1.2 (0.6)* | 0.1 (0.4) |
| Polecat | - | - | 1.2 (0.5)* |
| Stone marten | - | - | - |
| ***Ixodes hexagonus* larvae** |  |  |  |
| Badger | 3.3 (1.0)*** | 4.5 (0.9)*** | 2.2 (0.9)* |
| Pine marten | - | 1.2 (0.9) | -1.2 (0.7) **·** |
| Polecat | - | - | -2.4 (0.7)*** |
| Stone marten | - | - | - |
| ***Ixodes hexagonus* nymphs** |  |  |  |
| Badger | -1.2 (0.6)* | 1.1 (0.3)*** | -1.1 (0.5)* |
| Pine marten | - | 2.3 (0.6)*** | 0.1 (0.5) |
| Polecat | - | - | -2.2 (0.5)*** |
| Stone marten | - | - | - |
| ***Ixodes hexagonus* females** |  |  |  |
| Badger | -1.8 (0.7)* | 1.1 (0.3)*** | -1.5 (0.6)** |
| Pine marten | - | 2.9 (0.7)*** | 0.3 (0.7) |
| Polecat | - | - | -2.6 (0.6)*** |
| Stone marten | - | - | - |

**Additional file 1: Table S3.** Estimated odds ratio from outcomes generalized linear models. Model outcomes of generalized linear models with a binomial distribution and logit link function testing for differences in infection prevalence between feeding ticks of different stages found on all mustelids. Each cell gives the outcome of a single model. Estimated ln(odds ratio) and standard error (between brackets) are given for each combination. *P*-values are represented by **·** for *P* < 0.1, * for *P* < 0.05, ** for *P* < 0.01 and *** for *P* < 0.001.

| Difference | *B. burgdorferi* | *B. miyamotoi* | *A. phagocytophilum* | *N. mikurensis* |
| --- | --- | --- | --- | --- |
| *I. ricinus* nymphs vs adult | -0.6 (0.6) | -0.4 (1.1) | -0.9 (0.6) | 1.5 (0.5)** |
| *I. hexagonus* nymphs vs adults | -1.2 (0.5)* | - | -0.9 (0.2)*** | -0.2 (0.7) |
| *I. ricinus* vs *I. hexagonus* nymphs | 2.4 (0.7)*** | - | 0.9 (0.6) | 3.9 (0.6)*** |
| *I. ricinus* vs *I. hexagonus* adults | 1.9 (0.4)*** | - | 0.9 (0.2)*** | 2.2 (0.6)*** |

**Additional file 1: Table S4.** DNA sequences of *B. afzelii* (IGS) and *A. phagocytophilum* ecotype I (GroEL) from tissue samples.

>Spleen sample stone marten 162 (IGS *B. afzelii*)

cagggtacttagatggttcacttcccctggtatcgcctctattatttagataatagatagctagcatcttgctagctggattactccattcggtaatcttgggatcaataaatgtttgcttatcccccaagcttttcgcagcttaccacgaccttcttcgccttaaagctcctaggcatccaccatagactcttattactttgaccatatttttatcttccatctctattttgccaatttgtttatacaacatagaataatatatatctttgtttaatccatgtcaatatatatattattttttatattatttgaatgttttattcaaataatataaacatttaaaaaataaattcaaggtttaaagtataaaataaaaaccctggcaataacctactctcccgcgaact

>Spleen sample stone marten 0177 (IGS *B. afzelii*)

cagggtacttagatggttcacttcccctggtatcgcctctattatttagataatagatagctagcatcttgctagctggattactccattcggtaatcttgggatcaataaatgtttgcttttcccccaagcttttcgcagcttaccacgaccttcttcgccttaaagctcctaggcattcaccatagactcttattactttgaccatatttttatcttccatctctattttgccaatttgtttatacaacatagaataatatatatctttgtttaatccatgtcaatatatatattattttttatattatttgaatgttttattcaaataatataaacatttaaaaaataaattcaagatttaaagtataaaataaaaaccctggcaataacctactctcccgcgaact

>Liver sample polecat 187 (GroEL *A. phagocytophilum* ecotype I)

tgctgaaaaaatgctggtggaatttgaaaatccatacatattccttactgaaaagaagattaatcttgtacaaagcattctaccaatcttagaaaacgttgcacggtctggaagaccattgctcatcatagctgaagacgttgaaggtgaagctctgagcacgcttgtactcaataagctccgtggtggccttcaagttgctgctgtaaaggcgcctggtttcggtgacaggagaaaagacatgcttggcgatattgctgtaatagtaggcgctaagtatgtagtaaatgacgagcttgctgttaagatggaagacatcgctctaagcgatcttggtactgctaagagcgtacgcatcacaaaagacgcaactactatcataggtagtgttgatagcagttctgaaagcatagctagcaggactaatcaaatcaaagctcagatagaaaattctagttctgattatgacaaggaaaagcttagagaacgtttagcaaagct

>Liver sample polecat 285 (GroEL *A. phagocytophilum* ecotype I)

ggatatctttcgccttactttgttacaaatgctgaaaaaatgctggtggaatttgaaaatccatacatatttcttactgaaaagaagattaatcttgtacaaagcattctaccaatcttagaaaacgttgcacggtctggaagaccattgctcatcatagctgaagacgttgaaggtgaagctctgagcacgcttgtactcaataagctccgtggtggccttcaagttgctgctgtaaaggcgcctggtttcggtgacaggagaaaagacatgcttggcgatattgctgtaatagtaggcgctaagtatgtagtaaatgacgagcttgctgttaagatggaagacatcgctctaagcgatcttggtactgctaagagcgtacgcatcacaaaagacgcaactactatcataggtagtgttgatagcagttctgaaagcatagctagcaggactaatcaaatcaaagctcagatagaaaattctagttctgattatgacaaggaaaagcttagagaacgtttagcaaagct

>Liver sample polecat 409 (*A. phagocytophilum* ecotype I)

ggatatctttcgccttactttgttacaaatgctgaaaaaatgctggtggaatttgaaaatccatacatattccttactgaaaagaagattaatcttgtacaaagcattctaccaatcttagaaaacgttgcacggtctggaagaccattgctcatcatagctgaagacgttgaaggtgaagctctgagcacgcttgtactcaataagctccgtggtggccttcaagttgctgctgtaaaggcgcctggtttcggtgacaggagaaaagacatgcttggcgatattgctgtaatagtaggcgctaagtatgtagtaaatgacgagcttgctgttaagatggaagacatcgctctaagcgatcttggtactgctaagagcgtacgcatcacaaaagacgcaactactatcataggtagtgttgatagcagttctgaaagcatagctagcaggactaatcaaatcaaagctcagatagaaaattctagttctgattatgacaaggaaaagcttagagaacgtttagcaaagct
